# Household Drug Stockpiling and Panic Buying of Drugs During the COVID-19 Pandemic: A Study From Jordan

**DOI:** 10.3389/fphar.2021.813405

**Published:** 2021-12-22

**Authors:** Sura Al Zoubi, Lobna Gharaibeh, Hatim M. Jaber, Zaha Al-Zoubi

**Affiliations:** ^1^ Department of Basic Medical Sciences, School of Medicine, Al-Balqa Applied University, As-Salt, Jordan; ^2^ Pharmacological and Diagnostic Research Center, Faculty of Pharmacy, Al-Ahliyya Amman University, Amman, Jordan; ^3^ Department of Community Medicine, School of Medicine, Al-Balqa Applied University, As-Salt, Jordan; ^4^ Independent Researcher, Amman, Jordan

**Keywords:** COVID-19, drug shortages, panic buying, drug stockpiling, lockdown, drug management, Jordan

## Abstract

The coronavirus disease that emerged in 2019 (COVID-19) has affected health, societies and economies. Policies that have been imposed by different countries to slow the spread of the disease, including national lockdowns, curfews, border closures and enforcement of social distancing measures have disturbed the drug supply chain and resulted in drug shortages. Uncertainty concerning the pandemic has also led to the panic buying of drugs and the stockpiling of drugs in households, which has amplified the problem. In this cross-sectional study, a self-developed questionnaire was distributed online in order to a) assess the practice of household drug stockpiling prior to the national lockdown in Jordan, b) investigate the factors affecting it and c) measure peoples’ knowledge about the consequences of this behaviour. Results from this study show that drug purchasing was reported by 44.3% of the participants and was most common among participants from non-medical backgrounds (336, 75.7%) or those who have chronic diseases (261, 58.8%) and taking chronic supplements (282, 63.5%) regardless of their age, gender, living area or the possession of health insurance. Analgesics and antipyretics were the most frequently purchased drugs (225, 70.5%) and anticipation of their need was the most common reason for purchasing drugs (231, 52.0%). Buyers were also less aware, when compared to non-buyers, that panic buying and drug stockpiling may lead to drug shortages (204, 45.9% vs 325, 58.1%) and that this behaviour can pose a health hazard, especially to children (221, 47.5% vs 342, 61.2%). Our study shows that panic buying of drugs and household drug stockpiling were common in Jordan during the COVID-19 pandemic and this was related to participants’ medical knowledge and educational backgrounds. Therefore, educating the general population regarding rational drug use is urgently needed. This is also a compelling case for the development of national guidelines for drug management that target the general population and healthcare personnel, especially pharmacists, to avoid drug shortages during crises.

## Introduction

Since the start of the coronavirus pandemic (COVID-19), it has been affecting health, societies and economies (Zhou et al., 2020; Chen et al., 2021). Symptoms and manifestations of COVID-19 can be severe, with acute respiratory failure and sepsis that requires hospitalization, and sometimes even lead to death (Wiersinga et al., 2020). At the beginning of the pandemic, there was no FDA approved drug for the treatment of COVID-19, nor a vaccine to prevent and slow the spread of the disease.

Due to the lack of certainty regarding the treatment and prevention of the virus, and in order not to overwhelm the healthcare system, most countries imposed complete lockdowns. Other safety measures were also imposed, which included the enforcement of social distancing, such as curfews; working from home, reduced working hours for those who had to be at their place of work, reduced manpower, the closure of non-essential shops, and border closures along with the cancellation of non-essential travel. Applying these lockdowns and safety measures proved to be effective and resulted in the significant decline of the infection rate and the reduction in mortality, especially when such measures were applied early (Ghosal et al., 2020; Ji et al., 2020; Taghrir et al., 2020; Lau et al., 2021).

Although the FDA has approved the first drug, remdesivir, for certain patients (FDA, 2021) and several vaccines have also been approved for emergency use across the globe (WHO, 2021), many countries are still enforcing lockdowns and social distancing measures in order to alleviate the effect of the subsequent waves and the new variants of the virus.

Due to the lockdowns, many factories, including drug factories, were closed worldwide at the beginning of the pandemic or operated with a lower capacity (Bookwalter, 2021). China, which is considered the largest global supplier of active pharmaceutical ingredients (APIs), imposed a national lockdown in order to contain the virus. This was a significant concern for many countries, as China’s lockdown was thought to pose a risk to the supply chain of drugs and APIs, thereby resulting in a drug shortage (Chatterjee, 2020). Drug shortages are also a cause of reduced manpower, and other factors, such as factories being unable to work at maximum capacity and challenges to the importing of normal quantities due to transportation restrictions, and also the closure of ports, as well as increased demand due to panic buying.

Panic buying and stockpiling have affected many items, such as groceries, food, sanitisers, disinfectants and protective equipment, including face masks and gloves, around the world and have led to acute shortages for these items, leaving people in need with no means to obtain them. During this time, people might feel that the situation is out of their hands which generates many concerns, anxiety, and fear about resources insufficiency. Therefore, people act with their primitive instincts and tend to panic buy to mitigate this anxiety and fears (Arafat et al., 2020a). If such circumstances occur in regard to drugs, the consequences of drug shortages can be catastrophic for patient outcomes (Badreldin and Atallah, 2021). The balance between the drug supply and demand should always be maintained. This pandemic has already affected drug supplies due to the reduction of manufacturing. Therefore, an increase in the demand will exacerbate the problem, both in the short and the long term, and compromise the healthcare system’s ability to deal with this situation.

Patients, especially with chronic diseases, need to always have a supply of their respective drugs. Other drugs, such as pain killers and emergency drugs, also need to be available during lockdowns and curfews. People are encouraged to hold an adequate amount of essential drugs without buying excessively or stockpiling (American Pharmacists Association AS of H-SP and Foundation and NA of CDS, 2007). However, there is no clear guideline concerning how people should react during a pandemic in regard to keeping their supply of drugs without this affecting the volume of drugs in the supply chain and increasing the risk of stock-outs or keeping too many drugs at home.

Although the pharmaceutical industry in Jordan is considered to be one of the best in the region, Jordanian manufacturers export most of their production of drugs overseas and only a small percentage is left for the local market. This percentage is not sufficient for meeting the national demand, and therefore, most of the drugs that are used domestically in Jordan are still imported (Alomari et al., 2015; Jordan Chamber of Industry, 2021). The general population can obtain drugs depending on their medical and clinical needs, either as prescribed drugs through a prescription from healthcare providers, or through direct purchases of over-the-counter drugs from pharmacies. Despite the current national regulations that are stated in The Drug and Pharmacy Law No.12 which do not allow pharmacists to dispense prescription-only drugs without a prescription (Jordanian Ministry of Health, 2013), self-medication is a common practice (Yousef et al., 2008) and prescription-only drugs such as antibiotics can still be dispensed sometimes from pharmacies to patients who ask for them even without a prescription, which gives the general population open access to the majority of drugs classes, except controlled drugs (e.g., narcotics and psychotropic substances), with no limitations concerning the number of drugs that can be bought (Al-Husseini et al., 2018). Therefore, Jordan can be liable to drug shortages during crises such as wars, natural disasters, political conflicts and/or pandemics.

This study aims to assess the behaviour of the general population in Jordan in regard to the stockpiling and panic buying of drugs during the week before the full national lockdown due to the coronavirus pandemic, investigate the factors affecting it and measure their knowledge about the consequences of this behaviour.

## Methods and Materials

### Study Design, Population, and Sampling

This descriptive, online cross-sectional study was conducted at the beginning of the COVID-19 pandemic in Jordan, during the 3-day national full lockdown period. This study measured the behaviours of panic buying and drug stockpiling by the general population during the week before the full national lockdown in which the Jordanian government declared the state of emergency to limit the spread of COVID-19 and at the end of which a formal announcement about the lockdown was made. The study protocol is approved by the Research Ethics Committee of the School of Medicine at Al-Balqa Applied University.

The general population was targeted by this study. Eligibility criteria were being an adult (18 years or older) and living in the Hashemite Kingdom of Jordan. According to the Jordanian Department of Statistics and the real-time world statistic platform Worldometer, the estimated population of Jordan in 2020 was around 10,200,000. Of them, 55% are adults and 91.5% live in urban areas (Department of Statistics, 2021; Worldometer, 2021). Applying this information to calculate the minimum acceptable sample size using the Raosoft^®^ online sample calculator (Raosoft, 2021) with a response distribution of 50%, a confidence level of 99% and a margin of error of 5, the estimated sample size was 664. However, larger sample size was used to increase the power of our study.

A self-administered questionnaire was created using Google Forms and distributed online *via* different social media platforms. Participants were recruited *via* a convenient sampling method where participants were invited to voluntarily and anonymously complete and then submit their responses. The link to the questionnaire was available for 3 days only (the period of the full national lockdown) and all responses were considered for analysis.

### Questionnaire

After a comprehensive review of the literature, and due to the paucity of data and studies in this subject, a self-developed questionnaire was established and validated for this study depending on the aims of the study, the information required to be collected and the targeted population.

The questionnaire was developed in Arabic, and it consisted of 20 questions that were equally important and weighted the same. Questions were written simple, clear, short, and in a layperson language. All questions were close-ended with multiple choices and, depending on the question, participants were allowed to choose only one or more than one answer. Two of the questions had an open-ended choice to allow participants to elaborate upon their responses if they wish to. The questionnaire was divided into four sections to collect information concerning participants’ demography, social and health-related information, along with their practices, attitudes and knowledge concerning the subject of panic buying and the stockpiling of drugs during the crisis.

After the questionnaire was constructed, a panel of three experts in pharmacy, medicine and community medicine was formed to review the questionnaire for language or grammar mistakes, construction errors, or anything that might be considered unethical or offensive and then modifications were made where necessary. A pilot test was then carried out on 30 individuals who gave suggestions to improve a few questions and, at the same time, judged the face validity of the questionnaire. After the pilot run and the application of the final modifications, the panel re-assessed and evaluated the content validity of the questionnaire.

Before disseminating the questionnaire, A cover letter was attached to it that described the aims of this study, how responses would be submitted anonymously, and how data would be treated and analysed confidentially. There was also a statement that read: “Participation in this study is voluntary and by completing and submitting this form, participants are providing us with consent to use their data for this study.”

### Statistical Analysis

Data were analysed using IBM SPSS statistics (version 26) predictive analytics software. Descriptive analysis, including frequency and percentage, was used for the categorical variables. Chi-square *X* ^2^ was used to determine the level of significance between two or more variables. *p* values below 0.05 were considered significant.

## Results

### Participants’ Characteristics

One thousand and twenty (1,020) questionnaires were properly completed and 17 were excluded as one participant was not living in Jordan and 16 were younger than 18 years.

Out of the included 1,003 responses, 444 participants (44.3%) stated that they bought drugs during the week before the full national lockdown in which the Jordanian government declared the state of emergency to limit the spread of COVID-19 and at the end of which a formal announcement about the lockdown was made. Participants were from all age groups, both genders and lived across the Hashemite Kingdom of Jordan, but the decision to buy drugs was not affected by participants age, sex or living area (*p* > 0.05; Table 1). The majority of participants (961, 68.9%) were educated at the university/undergraduate level and were from a non-medical background (711, 70.9%), while a quarter of the participants were unemployed. All these elements significantly affected participants’ drug stockpiling habits (*p* < 0.05; Table 1).

**TABLE 1:** General characteristics of the participants.

	Buyers^a^(*n*= 444)	Non-buyers (*n* = 559)	Total (*n* = 1,003)	*p* Value
	*N* (%)	*N* (%)	*N* (%)	
Age				
18–25	216 (48. 6)	293 (52.4)	509 (50.7)	0.444
26–35	118 (26.6)	151 (27.0)	269 (26.8)	
36–45	67 (15.1)	69 (12.3)	135 (13.6)	
>45	43 (9.7)	46 (8.2)	89 (8.9)	
Gender				
Male	98 (22.1)	141 (25.2)	239 (23.8)	0.245
Female	346 (77.9)	418 (74. 8)	764 (76.2)	
Living area				
North	55 (12.4)	68 (12.2)	123 (12.3)	0.157
Middle	368 (82.9)	477 (85.3)	844 (84.2)	
South	21 (4.7)	14 (2.5)	35 (3.5)	
Education level				
School or diploma level	51 (11.5)	98 (17.5)	149 (14.9)	0.008
Undergraduate level	309 (69.6)	382 (68.3)	691 (68.9)	
Post graduate level	84 (18.9)	79 (14.1)	163 (16.3)	
Education background^b^				
Medical	108 (24.3)	184 (32.9)	292 (29.1)	0.003
Non-medical	336 (75.7)	375 (67.1)	711 (70.9)	
Occupation				
Student	180 (40.5)	231 (41.3)	411 (41.0)	0.045
Employed	173 (39.0)	182 (32.6)	355 (35.4)	
Unemployed	91 (20.5)	146 (26.1)	237 (23.6)	
Do you or any of your household members have chronic diseases that need chronic drug use?				
Yes	261 (58.8)	231 (41.3)	492 (49.1)	<0.001
No	153 (34.5)	299 (53.5)	452 (45.1)	
I don’t know	30 (6.8)	29 (5.2)	59 (5.9)	
Do you or any of your household members use drugs or supplements that are not related to chronic diseases?				
Yes	282 (63.5)	275 (49.2)	557 (55.5)	<0.001
No	125 (28.2)	236 (42.2)	361 (36.0)	
I don’t know	37 (8.3)	48 (8.6)	85 (8.5)	
Do you or any of your household members have medical insurance?				
I don’t know	5 (1.1)	12 (2.1)	17 (1.7)	0.637
No, none of us	91 (20.5)	119 (21.3)	210 (20.9)	
Yes, some of us	128 (28.8)	158 (28.3)	286 (28.5)	
Yes, all of us	220 (49.5)	270 (48.3)	489 (48.9)	

Among all participants, buyers were compared to non-buyers regarding their general characteristics. Data are presented as frequency (percentage) [N (%)]. Data were analysed using Pearson Chi-square tests. p values < 0.05 were considered statistically significant.

a Buyers are participants who bought drugs during the week before the full national lockdown.

b Medical background includes participants who study to become, or already are, medical doctors, dentists, clinical pharmacists, pharmacists, nurses, physiotherapists, or medical laboratory workers.

Almost half of the participants, or their household members, have chronic diseases (492, 49.1%) or take regular drugs or supplements that are not related to a chronic disease (557, 55.5%), and this significantly affected their decision to buy drugs or not (*p* < 0.05). Most participants were fully or partially covered by medical insurance. However, whether the participant had medical insurance or not did not influence the participants’ decision (*p* > 0.05; Table 1).

### Participants’ Decisions

**FIGURE 1:**
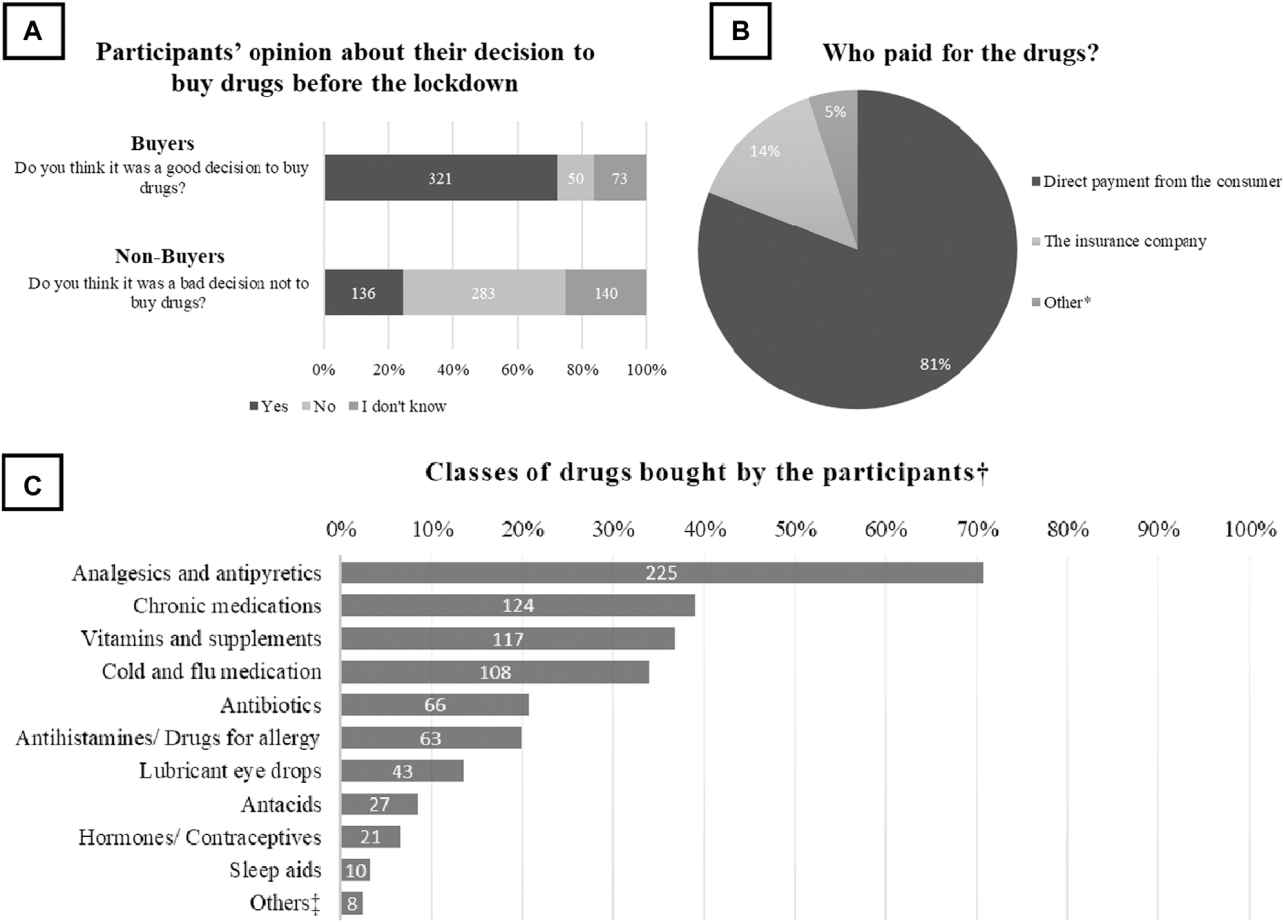
Characteristics of participants’ decisions. Participants were asked about **(A)** their opinion about the decision to buy drugs or not before the lockdown (*n* = 444 buyers and 559 non-buyers), **(B)** Who paid for the drugs they bought (*n* = 444) and **(C)** the classes of drugs they bought just in case they needed them during the lockdown (*N* = 318). *Free medical samples, government exemption of payment or medical aids, ^†^Classes of drugs purchased by people who were not in need at the time of the purchase ^‡^Laxatives, muscle relaxants and medical skin creams and ointments.

When the participants were asked about their opinion about the decision they took to buy or not to buy drugs before the lockdown, 50.6% (*N* = 283) of the non-buyers and 72.3% (*N* = 321) of buyers were happy about their decisions (Figure 1A). Among buyers, 80.9% (*N* = 359) paid directly to the pharmacy and the rest were either covered by their medical insurance and government exemption of payment or got free medical samples or medical aids (Figure 1B), and for those buyers who did not need the drug at the time of purchasing (*N* = 318), analgesics and antipyretics were the most frequently bought classes of drugs. (Figure 1C).

### The Effect of Educational Background on the Practice of Drug Purchasing and Stockpiling

Having a medical background did not affect participants’ behaviour in terms of the reasons behind drug purchasing, the number of drugs being purchased or the fate of the unused drugs (*p* > 0.05; Table 2). Although statistically insignificant, a higher percentage of participants from non-medical backgrounds stated that they will distribute the unused drugs to other people (17.0 vs 10.1%).

**TABLE 2:** Effect of educational background on the practice of drug purchasing and stockpiling.

	Medical^a^ (*n* = 108)	Non-medical (*n* = 336)	Total (*n* = 444)	*p* Value
	*N* (%)	*N* (%)	*N* (%)
The main reason behind buying the drugs before the lockdown			
Because it is time for a refill, or I need it now	30 (27.8)	96 (28.6)	126 (28.4)	0.249
In case I needed it during the lockdown	51 (47.2)	180 (53.6)	231 (52.0)	
Other^b^	27 (25.0)	60 (17.9)	87 (19.6)	
How long will the amount you bought be enough to meet your demand?			
Less than a week	19 (17.6)	38 (11.3)	57 (12.8)	0.203
From a week to a month	69 (63.9)	232 (69.0)	301 (67.8)	
More than a month but less than 3 months	16 (14.8)	60 (17.9)	76 (17.1)	
For 3 months or more	4 (3.7)	6 (1.8)	10 (2.3)	
How often do you buy this number of drugs?			
Always	50 (46.3)	123 (36.6)	173 (39.0)	0.004
Sometimes	48 (44.4)	133 (39.6)	180 (40.5)	
Rarely or never	10 (9.3)	80 (23.8)	91 (20.5)	
The fate of the unused drugs after the lockdown is over			
I will keep them until their expiry date	87 (80.6)	240 (71.4)	327 (73.7)	0.151
I will give them to my friends, relatives or people who need them	11 (10.1)	57 (17.0)	68 (15.3)	
I still don’t know	10 (9.3)	39 (11.6)	49 (11.0)	

Among participants who bought drugs during the week before the lockdown, participants from medical backgrounds were compared to participants from non-medical backgrounds regarding their practice of drug purchasing and stockpiling. Data are presented as frequency (percentage) [N (%)]. Data were analysed using Pearson Chi-square tests. p values < 0.05 were considered statistically significant.

a Medical background includes participants who study to become, or already are, medical doctors, dentists, clinical pharmacists, pharmacists, nurses, physiotherapists, or medical laboratory workers.

b Participants stated that they bought drugs before the lockdown to give them to people who might need them or because they were afraid of drugs shortages after the lockdown.

### Attitudes Towards the Stockpiling of Drugs

Participants’ responses were analysed twice in order to measure the effect of their knowledge concerning their attitudes and the effect of educational background on their understanding of the issues.

There was no statistically significant difference between buyers and non-buyers (18.9 vs 21.8%) in terms of their attitudes concerning self-medication for the treatment of COVID-19 (*p* > 0.05). However, a significantly higher percentage of participants from medical backgrounds were against self-medication (*p* < 0.05; Table 3).

**TABLE 3:** Participants’ knowledge about self-medication with COVID-19 drugs and risk of drug stockpiling.

	Total (n = 1,003)					
	Buyers (n = 444)	Non-buyers (n = 559)	*p* Value	Medical (n = 292)	Non-medical (n = 711)	*p* Value
	N (%)	N (%)		N (%)	N (%)	
Are you willing to buy a drug to self-treat COVID-19 at home?						
Yes	84 (18.9)	122 (21.8)	0.246	42 (14.4)	165 (23.2)	<0.001
No	252 (56.8)	288 (51.5)		184 (63.0)	356 (50.1)	
Maybe	108 (24.3)	149 (26.7)		66 (22.6)	190 (26.7)	
Do you think that stockpiling of drugs in the household at this stage will lead to drug shortages later?						
Yes	204 (45.9)	325 (58.1)	0.001	157 (53.8)	372 (52.3)	0.519
No	92 (20.7)	84 (15.0)		45 (15.4)	131 (18.4)	
Maybe	148 (33.3)	150 (26.8)		90 (30.8)	208 (29.3)	
Do you think that stockpiling of drugs in the household can pose a health hazard especially on children?						
Yes	211 (47.5)	342 (61.2)	<0.001	167 (57.2)	386 (54.3)	0.255
No	103 (23.2)	76 (13.6)		43 (14.7)	136 (19.1)	
Maybe	130 (29.3)	141 (25.2)		82 (28.1)	189 (26.6)	

Among all participants, buyers were compared to non-buyers and participants from medical backgrounds were compared to participants from non-medical backgrounds regarding their knowledge about self-medication with COVID-19 drugs and the risk of drug stockpiling. Data are presented as frequency (percentage) [N (%)]. Data were analysed using Pearson Chi-square tests. p values <0.05 were considered statistically significant. Buyers are participants who bought drugs during the week before the full national lockdown. Medical background includes participants who study to become, or already are, medical doctors, dentists, clinical pharmacists, pharmacists, nurses, physiotherapists, or medical laboratory workers.

Knowledge regarding the consequences of purchasing drugs and stockpiling was not influenced by participants’ medical backgrounds (*p* > 0.05). However, these practices were significantly affected by participants’ knowledge, with a higher percentage of non-buyers being aware that drug stockpiling would create the risk of drug shortages and/or harm to children. (*p* < 0.05; Table 3).

## Discussion

Almost half of the participants in our study stockpiled drugs just prior to the lockdown. In such cases, the drugs were mainly used for the management of chronic conditions, supplements, antibiotics, analgesics. In a country such as Jordan, with limited resources and a high dependence on other countries for the supply of generic drugs or raw materials, a sudden increase in demand within the local market amid international restrictions on trade will jeopardize the availability of drugs. This behaviour was irrelevant to participants’ age, gender, living area or educational level. However, in a study conducted in Brazil, male gender, higher socioeconomic status and younger age were correlated with a higher prevalence of panic buying (Lins and Aquino, 2020). The effect of socio-economic status on the patterns of pharmaceutical panic buying was also revealed in a study that was conducted by Elek et al. in Hungary. The findings of the study showed that socioeconomic status affected the patterns of pharmaceutical panic buying. People with limited income were much less likely to stockpile pharmaceutical products, which was a result of having modest and/or inadequate finances and reduced access to pharmacies and physicians (Elek et al., 2021).

Findings from our study show a higher percentage of people from non-medical backgrounds rarely stockpile drugs in normal conditions compared to people from a medical background. However, the percentage of buyers was significantly higher than non-buyers for participants with a non-medical background. This implies that these drugs were not part of the health care plan for people from non-medical backgrounds, though they were purchased during the pandemic. This, therefore, suggests the importance of medical knowledge in determining the behaviour of consumers during a crisis. In Indonesia, a study conducted on people who practised panic buying during COVID-19 concluded that knowledge is a key factor affecting this behaviour (Wijaya, 2020). Results from this study along with our results suggest that educating the population could attenuate this phenomenon.

Consumers from non-medical backgrounds are easily influenced by traditional and/or social media, concerns from being infected with the coronavirus, or rumours of the possible efficacy of certain drugs/supplements, and the possibility of lockdowns. In a study, Arafat and others collected information from traditional media reports about panic buying during the early stages of COVID-19 and showed that there was a high abundance of reports that mentioned panic buying and showed photos of empty shelves (Arafat et al., 2020b). Such anxieties disseminate *via* media and stimulate consumers from non-medical backgrounds to stockpile drugs, which subsequently cause them to be more positive to the idea of self-medication with COVID-19 drugs and supplements if they could acquire them as shown in our results. A similar case was reported in China where a woman who did not have COVID-19 self-medicated with hydroxychloroquine sulfate and was admitted to the intensive care unit suffering from cardiac arrhythmias (Liu et al., 2020). Therefore, during the pandemic, people required accurate, reliable information from trusted organizations that can be easily accessed (Aslani, 2020). Pharmacists can serve as educators concerning public health issues and serve as impeccable sources of knowledge. Moreover, they can provide remote pharmaceutical care services for patients that can reassure patients, reduce their risk of being infected with the disease, and monitor and report drug shortages (Liu et al., 2020).

The most purchased drugs were analgesics and antipyretics, followed by chronic medications, and vitamins and supplements. However, in Germany, the surge in purchasing drugs during the pandemic was highest for anti-Parkinson’s disease drugs and tranquillizers (Kostev and Lauterbach, 2020). In Australia, several reports showed that pharmacists observed increased purchases of thyroxine, paracetamol and hydroxychloroquine among other drugs (Bell et al., 2020). Similarly, a study from Hungary showed that at the beginning of the pandemic there was an increase in demand on almost all drugs classes especially gastrointestinal drugs (Elek et al., 2021).

Our study revealed that 81% of the consumers paid themselves for the drugs, though most of them were covered by medical insurance. This demonstrates a high degree of willingness to purchase drugs even during an economically challenging condition. This behaviour can be interpreted as a coping mechanism during stressful conditions in order to maintain the health of one’s family or to act in a fashion similar to other members of the community (Kostev and Lauterbach, 2020; Yuen et al., 2020; Chua et al., 2021).

An important finding from our study is that 73.7% of the buyers stated that they will keep unused drugs in the household until the expiry date passes, despite their knowledge of the safety concerns that this might pose if drugs were not stored or disposed of properly. A cross-sectional study from Jordan by Naser et al. shows that most households in Jordan inappropriately deal with the drugs in their household in terms of storage or disposal (Naser et al., 2021).

The major strength of our study is its originality. Due to the lack of similar studies, this research would be a backbone for future studies that highlight such important issues in such challenging times. On the other hand, our study has certain limitations including the small number of participants and the fact that panic purchasing of drugs was studied from the patients’ perspective only. With this in mind, future studies must be carried out with a larger sample size and from the pharmacists’ perspective, too. In addition, investigation of drug shortages during the pandemic will shed more light on the different aspects of the problem and possibly establish a clear connection to the panic buying of drugs during the same period.

## Conclusion

The coronavirus pandemic has been a unique experience that revealed the vulnerability of humans in front of this dangerous and universal threat. The desire to secure drugs in case of a lockdown, the prospect of drug shortages and possible infection of COVID-19, caused consumers to buy excess amounts of drugs in order to feel secure, which served as a coping mechanism during the stressful conditions emanating from the pandemic. Pharmacists must play a role in providing alternatives for patients in case of drug shortages, which should comprise remote pharmaceutical care services and the provision of correct information and knowledge concerning the circulated material available *via* social media. National guidelines for drug management, drug security and rational drug use during crises that target both consumers and pharmacists, are urgently needed and should be available for future needs. Additionally, it is necessary to impose stricter measures to increase compliance with The Drug and Pharmacy Law that controls the drug dispensing from pharmacies to promote rational drug use.

## Data Availability

The original contributions presented in the study are included in the article/Supplementary Material, further inquiries can be directed to the corresponding author.
